# Soy-Derived Agroindustrial Byproducts as Sustainable Substrates for Biohydrogen Production via Dark Fermentation

**DOI:** 10.1021/acsomega.5c09009

**Published:** 2026-01-30

**Authors:** Marcela Moreira Albuquerque, Gabriela de Bona Sartor, Thamarys Scapini, Walter Jose Martinez-Burgos, Thiago Edwiges, Carlos Ricardo Soccol, Adriane Bianchi Pedroni Medeiros

**Affiliations:** † 28122 Federal University of Parana, Department of Bioprocess Engineering and Biotechnology, R Francisco H. dos Santos, Centro Politécnico, Curitiba 81531-990, PR, Brazil; ‡ 74354 Federal University of Technology—Paraná, R. Deputado Heitor Alencar Furtado, 5000, Curitiba 81280-340, PR, Brazil

## Abstract

The valorization of byproducts from biofuel production is crucial in advancing sustainable energy solutions. Particularly in biodiesel processes, byproducts such as glycerin, soapstock, and wastewater with high Chemical Oxygen Demand (COD) and Biological Oxygen Demand (BOD) contain rich organic content suitable for biological conversion. Among these, soybean molasses and soapstock stand out as cosubstrates for dark fermentation to produce biohydrogen. This study aimed to obtain a new microbial consortium from the sludge of an anaerobic reactor thermally pretreated at 80 °C for 60 min and its use in bioH_2_ production in a culture medium composed of soybean molasses diluted in a pretreated lysogoma. All experiments were conducted at bench scale under batch conditions. The physicochemical parameters (soybean molasses concentration, pH, and temperature) were optimized using Central Composite Rotatable Design (CCRD) and response surface analysis. The profiles of bioH_2_ production, sugar consumption, and volatile fatty acid production were evaluated through kinetic analysis under optimal conditions. The kinetic analysis of the points 0, 26, and 54 h was also used to verify the consortium’s behavior throughout dark-fermentation. *Clostridium butyricum* was the predominant species in the initial consortium and throughout fermentation, indicating the success of the inoculum’s thermal pretreatment method, which resulted in its enrichment with spores of this microorganism, widely used in dark-fermentation trials. Sucrose was consumed as the preferred sugar, yielding acetic and butyric acids as fermentation byproducts. The maximum bioH_2_ production was achieved after 48 h of fermentation, resulting in a gas composed of H_2_ (57.14%) and CO_2_ (42.86%), culminating in a yield of 1.6 L of bioH_2_/L of culture medium. These findings underscore the viability of soybean byproducts as sustainable feedstocks for biological hydrogen production, contributing to circular bioeconomy strategies and renewable energy development.

## Graphical abstract

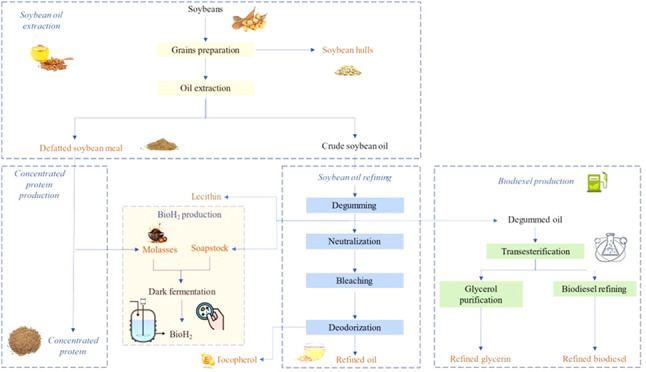

## 1 Introduction

Soybeans (*Glycine max* (L.) Merr) are one of the oldest and most relevant agricultural practices, reaching a global production of about 42,078 million tons in the 2024/2025 harvest, with Brazil and United States, being the largest producers, with 169 Mt, and 118 Mt, respectively.^1^ Approximately 85% of the entire production is directed to crushing, to obtain soybean meal and oil.^2^ About 57% of the total oil production is intended for human consumption, while approximately 16% is used for biodiesel production. The remainder used in the manufacture of cosmetics, varnishes, and animal feed.^3,4^

Crude soybean oil is composed, in addition to triacylglycerols, of free fatty acids (FFA), phospholipids, volatile/odorous components, dark pigments, and moisture. These elements trigger its oxidative deterioration, impacting the quality of the oil intended for food and hindering transesterification and glycerin separation during biodiesel production.^5^ These contaminants are removed through a refining process, resulting in the formation of high amounts of wastewater, commonly called sludge or soapstock, which represents about 1–6% of the total crude oil refined.^6^ Lysogoma is a residue generated during the biodiesel manufacturing process. Specifically, it is formed in the enzymatic pretreatment stage of soybean oil to remove undesirable components.

In addition to wastewater, the soybean processing chain also results in the production of protein concentrate, lecithin, tocopherol, molasses, and soybean hulls, mainly destined for human consumption and poultry or ruminant livestock feed.^7^ Soy molasses, an abundant byproduct of protein concentrate production, is characterized by its low added value and rich composition of fermentable sugars such as fructose, glucose, sucrose, stachyose, and raffinose^8^ as well as lipids, proteins, fibers, and ash.^9^ Its composition makes it a potential substrate for microbial oxidation in fermentative processes.^10^

The growing demand for products derived from soybean processing, in addition to the need for economic valorization of its byproducts and residues such as molasses and soybean lysogoma to improve the sustainability and circularity of its production chain makes the soybean chain a potential source of substrates for obtaining biofuels. Hydrogen (H_2_) is a sustainable biofuel with a high energy content (122 kJ/g), at least 2.75 times higher than hydrocarbon fuels^11^ and releases water vapor as the only byproduct of its combustion,^12^ making it constantly cited as the energy vector of the future.^13^ However, the H_2_ economy relies on the development of environmentally friendly production technologies, as its current demand is mainly met through energy-intensive thermochemical processes that release atmospheric pollutants and intensify the greenhouse effect.^14^ Thus, its production through biological routes such as dark fermentation constitutes a potential alternative. Besides the simplicity and operability in mild conditions, this process also allows for lower energy expenditure and nutrient recycling,^12^ although it is important to constantly explore new microorganisms and optimize physicochemical parameters to maximize production.

Microorganisms belonging to the class *Clostridia*, composed of strictly anaerobic bacteria such as those of the genus *Clostridium* are widely used in dark fermentation assays due to numerous literature reports on their excellent performance in bioH2 production.^13^ These microorganisms form protective spores, resistant to high temperatures and extreme acidity and alkalinity, a characteristic that can be exploited in obtaining consortia pretreated by physicochemical methods that favor their predominance and eliminate or drastically reduce the presence of methanogens, possibly resulting in higher amounts of bioH_2_.^15^ According to the physicochemical compositions of soybean lysogoma and soy molasses, their combination in the formulation of a culture medium for bioH_2_ production by dark fermentation may present synergistic effects, as the high viscosity and concentration of molasses require dilution for its use in fermentative processes, allowing its combination with wastewater from industrial processes.^16^ Furthermore, no studies using sludge or lysogoma in dark fermentation processes have been reported to date.

This study aimed to explore for the first time the potential of bioH_2_ production by dark fermentation through the combination of an aqueous fraction of lysogoma and soybean molasses as substrates, using a new consortium mainly composed of *Clostridium butyricum*. The optimization of physicochemical parameters such as soy molasses concentration, pH, and temperature were performed using the central composite rotational design (CCRD) and response surface methodology. The bioH_2_ production profile and volatile fatty acids were evaluated through kinetic analysis under optimal conditions and the consortium’s behavior was observed through metagenomic analysis.

## 2 Materials and Methods

### 2.1 Origin of Lysogoma and Molasses

Lysogoma was kindly donated by a soybean oil biodiesel company located in Lapa, Brazil (25°43′19.2″S 49°44′13.4″W). The lysogoma was collected in 5 L containers and stored at −20 °C until its use. Crude lysogoma was characterized according to the analytical methods described in Section 2.7. The soy molasses was kindly donated by a company located in Araguari, Brazil (18°40′51.4″S 48°09′20.1″W) and stored at room temperature until its use.

### 2.2 Pretreatment of Crude Lysogoma

Crude lysogoma was pretreated to recover its free fatty acids resulting from the soybean oil refining. This is a crucial step to turn crude lysogoma into a useful substrate for fermentation process.^17^ Based on previous assays, lysogoma was subjected to thermal pretreatment in an autoclave at 121 °C and 1 atm for 15 min. The heat, combined with its acidic characteristics, partially hydrolyzes or removes the remaining ester bonds of triglycerides and phosphatides, resulting in the destruction of its emulsion and subsequent spontaneous separation into two phases, one aqueous and the other oily. The oil layer is called acid oil, which contains free fatty acids (FFA) and glyceride. The lysogoma was cooled to room temperature, and the aqueous phase was removed using a 50 mL syringe. After separating the oily phase, the aqueous phase was centrifuged for 5 min at 2000 rpm to completely remove residual lipids and stored at −20 °C until used in the culture medium as a diluent.

### 2.3 BioH_2_-Producing Consortium

A bioH_2_-producing consortium was obtained from the sludge of the anaerobic reactor provided by a biodiesel company located in Lapa, Brazil. The sample was collected in a sterile bottle and then subjected to thermal pretreatment at 80 °C for 60 min in a water bath to inhibit methanogenic microorganisms.^18^

The pretreated sample was cooled to room temperature, and approximately 0.1 g (≅17 g/L) was added to a Hungate tube containing 6 mL of modified RCM (Reinforced Clostridial Medium) culture medium, consisting of 10 g/L yeast extract, 10 g/L meat extract, 10 g/L peptone, 10 g/L glucose, and a micronutrient solution suggested by^19^ containing 1 g/L ammonium chloride (NH_4_Cl), 2 g/L sodium chloride (NaCl), 0.5 g/L magnesium chloride (MgCl_2_·6H_2_O), 0.05 g/L calcium chloride (CaCl_2_·2H_2_O), 1.5 g/L dibasic potassium phosphate trihydrate (K_2_HPO_4_·3H_2_O), 0.75 g/L monopotassium phosphate (KH_2_PO_4_), and 2.6 g/L sodium bicarbonate (NaHCO_3_).

The pH of the culture medium was adjusted to 6.0 ^20^ with a 5 M HCl solution. The tube was purged with nitrogen gas at a flow rate of 10 mL/min for 3 min to remove oxygen and ensure anaerobiosis, then sealed with a Bakelite screw cap and incubated in an oven at 37 °C for 72 h. To assess bioH_2_ production by the consortium, the gas produced in the synthetic medium was collected and analyzed by gas chromatography. Subsequently, the consortium was transferred weekly to a fresh culture medium following the same conditions described above for maintenance purposes.

### 2.4 Consortium Molecular Identification and Bioinformatics Analysis

The consortium obtained from the anaerobic reactor underwent molecular analysis to determine its potential variation throughout the fermentation process was also evaluated during kinetic analysis (0, 26, and 54 h) under optimized conditions for bioH_2_ production. Fermented samples of approximately 6 mL were homogenized, and 1 mL was used for total DNA extraction. Subsequently, the 16S gene was amplified and sequenced using the Illumina NextSeq platform, and the sequences were analyzed with Qiime software to identify the microorganisms present in the samples as well as their respective percentages (Table 1).^24^

**Table 1:** Experimental Independent Variables and Related Levels Coding

independent variables	concentration (g_soy molasses_/L_lysogome_)	temperature (°C)	pH
–1.68	12.7	19.2	5.5
–1	40	26	6.2
0	80	36	7.2
1	120	46	8.2
1.68	147.3	52.8	8.9

### 2.5 Experimental Design, Optimization, and Statistical Analysis

A central composite rotational design (CCRD) with three factors and five levels (Table 2) was used to predict the experimental effects and interactions on bioH_2_ yield obtained from a medium composed of soybean molasses and the aqueous fraction of pretreated lysogoma, which was used as a diluent. The independent variables studied were based on the practical range reported in the scientific literature: soy molasses concentration between 12.7 to 147.3 g_molasses_/L_medium_, temperature between 19.2 to 52.8 °C, and initial pH between 5.5 to 9.9.^21,22^

**Table 2:** Physicochemical Characteristics of Raw Soybean Lysogoma (Untreated Thermally)

parameter	value
pH	4.6
biochemical oxygen demand (BOD)	163.33 g/L
chemical oxygen demand (COD)	287.6 g/L
soluble chemical oxygen demand	48.33 g/L
mineral oils and greases	1.40 g/L
total oils and greases	38.82 g/L
oils and vegetable fats/animal fats	37.41 g/L
Kjeldahl nitrogen	0.02 g/L
total nitrogen	0.13 g/L
total dissolved solids (TDS)	75.44 g/L
total suspended solids (TSS)	50.68 g/L
ash (%)	0.81%
total organic carbon	3.9 g/L
carbon/nitrogen ratio	30.2
total solids (TS)	126.12 g/L
total solatile Solids (TVS)	118.02 g/L
glucose	3.1 g/L
acetic acid	1.51 g/L
propionic acid	1.62 g/L
butyric acid	0.81 g/L
Na^+^	9.07 g/L
NH_4_^+^	0.03 g/L
K^+^	0.23 g/L
Mg^+^	0.47 g/L
Ca^+^	0.49 g/L
F^–^	0.3 g/L
Cl^–^	1 g/L
Br^–^	0.3 g/L
PO_4_^–^	>6.73 g/L
SO_4_^–^	>14.04 g/L

Nineteen experiments were conducted, including 8 factorial points, 6 axial points, and 4 central points, evaluating the bioH_2_ yield in mL/g of soy molasses (SM) after 48 h of fermentation as the response variable. All analyses were performed using Statistica software (version 7.0).

The experiments were carried out in 16 mL Hungate tubes containing 5.4 mL of culture medium composed of aqueous fraction from pretreated lysogoma and soy molasses, with pH adjusted using 5 M KOH and 5 M HCl solutions. The tubes were then purged with N_2_ gas for 40 s to ensure anaerobic conditions, sealed with Bakelite screw caps, and autoclaved at 121 °C for 15 min. Subsequently, the tubes were inoculated with 10% inoculum (v/v)^23^ and incubated in an oven for 48 h. After the incubation period, the gas volume was measured and analyzed by gas chromatography to determine its composition.

Following the linear regression analysis of the experimental results from the CCRD, nonsignificant terms were excluded, and a second-order polynomial regression model was constructed to express the relationship between independent and dependent variables, as shown in eq 1, where *Y* is the predicted value of bioH_2_ in mL/gSM, *X_i_* and *X_j_* are the independent variables, β_0_ is the model constant, β*_i_* are the linear coefficients, β*_ii_* are the quadratic coefficients, and *β_ij_* are the interaction coefficients.

1 $$Y=\beta_{0}+\sum \beta_{i}X_{i}+\sum \beta_{\mathrm{ii}}X_{i}^{2}+\sum \beta_{\mathrm{ij}}X_{i}X_{j}$$

Later, the model was validated (*p* < 0.1) through ANOVA, and response surfaces and contour plots were generated. The mathematical model was also used to obtain the optimal conditions for bioH_2_ production. Based on the mathematical model obtained, optimal values for molasses concentration, initial pH, and temperature for bioH_2_ production were determined and experimentally validated to confirm the model.

### 2.6 Kinetic Analysis of bioH_2_ and Organic Acids Production

The kinetic analysis experiments were conducted under optimal conditions, following the same cultivation conditions described for the central composite rotational design (CCRD) experiment. Substrate consumption, bioH_2_ production, and volatile fatty acids quantification were carried out every 6 h for 48 h, starting from the onset of bioH_2_ production (26 h). Fermented samples from each kinetic point (0, 26, 30, 36, 42, and 48 h) were prepared by centrifugation at 3000 rpm for 10 min to remove biomass, dilution, and microfiltration using cellulose acetate membranes (0.22 μm), then analyzed by HPLC.

### 2.7 Analytical Methods

The analyses of BOD (biochemical oxygen demand), COD (chemical oxygen demand), soluble COD, mineral oils and greases, total mineral oils and greases, vegetable oils and greases, animal fats, total Kjeldahl nitrogen, total nitrogen, total dissolved solids (TDS), ashes, total organic carbon, sulfates, phosphates, total solids (TS), and volatile total solids were performed according to the Standard Methods for the Examination of Water and Wastewater.^24^

Gas measurement and collection in the bioH_2_ production experiments were carried out using a special glass syringe for gases (Arti Glass, 100 mL). Gas composition was analyzed using a Micro GC System 490 gas chromatograph (Agilent) equipped with two columns (Molsieve 5 Å and PoraPLOT U) and a thermal conductivity detector (TCD). For the Molsieve 5 Å column, the operating parameters were: injection temperature of 110 °C, injection time of 20 ms, column temperature of 90 °C, and initial pressure of 190 kPa. Regarding the PoraPLOT U (PPU) column, the injection temperature was maintained at 110 °C, with a column temperature of 90 °C, and an initial pressure of 150 kPa. Each run was performed in a time of 1.2 min. Argon was used as the carrier gas, with a purity of 99.99%.

Sugars and volatile fatty acids were analyzed using a high-performance liquid chromatography (HPLC) system Shimadzu LC-20AD equipped with an Aminex HPX-87H column (Bio-Rad). The column was stabilized at 65 °C and eluted with 10 mmol L^–1^ of H_2_SO_4_ at a flow rate of 0.6 mL min^–1^. Sample injection was 20 μL, and components were detected by differential refractometry (Shimadzu RID-10 A).

Anions were analyzed by ion chromatography (761 Compact IC, Metrohm AG). Metrosep C 3250/4.0 and Metrosep A Supp 5–250/4.0 columns were used for cations and anions, respectively. For cations, the mobile phase was 3.5 mM HNO_3_ at a flow rate of 0.9 mL/min. For anions, the mobile phase used was 3.2 mM Na_2_CO_3_ and 1.0 mM NaHCO_3_ with a flow rate of 0.7 mL/min. Run times were 25 and 30 min for cations and anions, respectively.

## 3 Results and Discussion

### 3.1 Soybean Molasses and Lysogoma Characterization

The soybean molasses (SM) had a pH of 4.6 and it was composed of 250 g/kg moisture and 26, 199, 50, 119, and 6 g/kg fructose, sucrose, raffinose, stachyose and glucose, respectively. The complexity of the lysogoma can be noted by the characterization presented in Table 3, where a high BOD, COD, TDS, and TSS can be observed. Additionally, a high concentration of sulfates, phosphates, free fatty acids, triglycerides, and phospholipids is expected in the soybean lysogoma composition, with average values dependent on the raw material composition and industrial operations involved.^25^ Another noticeable characteristic of lysogoma is its acidic pH (4.6) resulting from the acidulation process by adding sulfuric acid to achieve partial acid hydrolysis or removal of ester linkages of remaining triglycerides and phospholipids in the soybean lysogoma, thereby facilitating phase separation (aqueous and oily).^26^

**Table 3:** BioH_2_ Production Results Obtained from the Central Composite Rotational Design after 48 h of Incubation^a^

	coded			real			
	soybean molasses concentration	temperature	pH	soybean molasses concentration g/L	temperature °C	pH	BioH_2_ yield mL/g SM
1	1	–1	–1	120	26	6.2	0
2	1	–1	1	120	26	8.2	7.61
3	1	1	–1	120	46	6.2	0
4	1	1	1	120	46	8.2	0
5	–1	–1	–1	40	26	6.2	8.06
6	–1	–1	1	40	26	8.2	22.47
7	–1	1	–1	40	46	6.2	0
8	–1	1	1	40	46	8.2	0
9	–1.68	0	0	12.7	36	7.2	13.91
10	1.68	0	0	147.3	36	7.2	10.26
11	0	1.68	0	80	52.8	7.2	0
12	0	–1.68	0	80	19.2	7.2	0
13	0	0	–1.68	80	36	5.52	0
14	0	0	1.68	80	36	8.9	16.38
15	0	0	0	80	36	7.2	23.26
16	0	0	0	80	36	7.2	23.01
17	0	0	0	80	36	7.2	23.05
18	0	0	0	80	36	7.2	20.32

a SM: Initial soybean molasses.

The oil layer of the lysogoma is called fatty acid oil, which contains free fatty acids (FFAs) and glycerides, typically sold as cattle feed for a low commercial value.^6^ Meanwhile, the aqueous phase is directed to the company’s wastewater treatment plant. As an alternative, the aqueous phase of lysogoma can be valorized as a diluent substrate for dark-fermentation process, besides serving as a nitrogen source, essential for nucleic acid, protein, and enzyme formation necessary for bioH_2_ production, and a phosphate source, crucial for medium buffering, lipid bilayer production, and energy in the form of adenosine triphosphate (ATP).^27^ Furthermore, other micronutrients such as Na^+^, NH4^+^, K^+^, Mg^+^, Ca^+^, F^–^, Cl^–^, Br^–^ which are essential for cell growth are also present in the lysogoma.^28^

### 3.2 Experimental Design, Optimization, and Statistical Analysis

The CCRD matrix and the bioH_2_ yields resulting from each trial can be observed in Table 3.

Through the results obtained in the CCRD assays, it was possible to analyze the significant terms for the process using linear regression analysis (Table 4), with a confidence interval of 90%. P-values less than 0.1 indicate that the terms of the model are significant for the process.^29^ Except for the interaction term between carbon and pH, all linear and quadratic terms were significant.

**Table 4:** Linear Regression Analysis of the Experimental Data Obtained through CCRD

	*R* ^2^ = 92.59; adj: 85.99			
factor	regression coefficient	standard error	*t*-value	*P*-value^a^
intercept	22.460	1.832	11.82095	0.000002
(1) soybean molasses (L)	–2.129	0.994	–2.06320	0.072997
soybean molasses (Q)	–3.877	1.033	–3.62599	0.006724
(2) temperature (L)	–2.795	0.993	–2.71170	0.026588
temperature (Q)	–8.159	1.033	–7.61458	0.000062
(3) pH (L)	3.630	0.993	3.52532	0.007785
pH (Q)	–5.257	1.033	–4.90753	0.001182
1L by 2L	2.865	1.033	2.12939	0.065855
2L by 3 L	–2.752	1.298	–2.04589	0.074985

a Significance level: 0.1.

The significant terms (*p* ≤ 0.1) were used to construct a second-order polynomial model (eq 2). The coefficient of determination *R* ^2^ was 0.9259, indicating that the model explains 92.59% of the response variable’s behavior within the evaluated range.

2 $$\mathrm{mL bio}H_{2}/\mathrm{gSM}=22.46-2.13\cdot x_{1}-3.88\cdot x_{1}^{2}-2.79\cdot x_{2}-8.15\cdot x_{2}^{2}+3.63\cdot x_{3}-5.26\cdot x_{3}^{2}+2.86\cdot x_{1}\cdot x_{2}-2.75\cdot x_{2}\cdot x_{3}$$

The variable codes in the model equation correspond to g/L of soybean molasses (*x*_1_), temperature (*x*_2_), and initial pH (*x*_3_). Subsequently, the mathematical model was validated through ANOVA, indicating a calculated *F* (14.0) greater than the tabulated *F* (2.47). After this validation, response surface and contour plots were generated. The lack-of-fit test was also evaluated, with the calculated *F* value (9.87) being higher than the tabulated *F* (5.28) at the 90% confidence level (p 0.0437). This result suggests that, although the model explains most of the variability (*R* ^2^ 92.59%), the lack of fit is statistically significant, indicating a small divergence between the model and the experimental data within the studied range, which is expected in complex biological systems such as the one evaluated. Nevertheless, the model showed good predictive capability, adequately explaining the observed variability. After this validation, response surface and contour plots were generated. The contour plots (Figure 1) show optimal bioH2 production across a wide range of pH, temperature, and concentration of soybean molasses.

**Figure 1:**
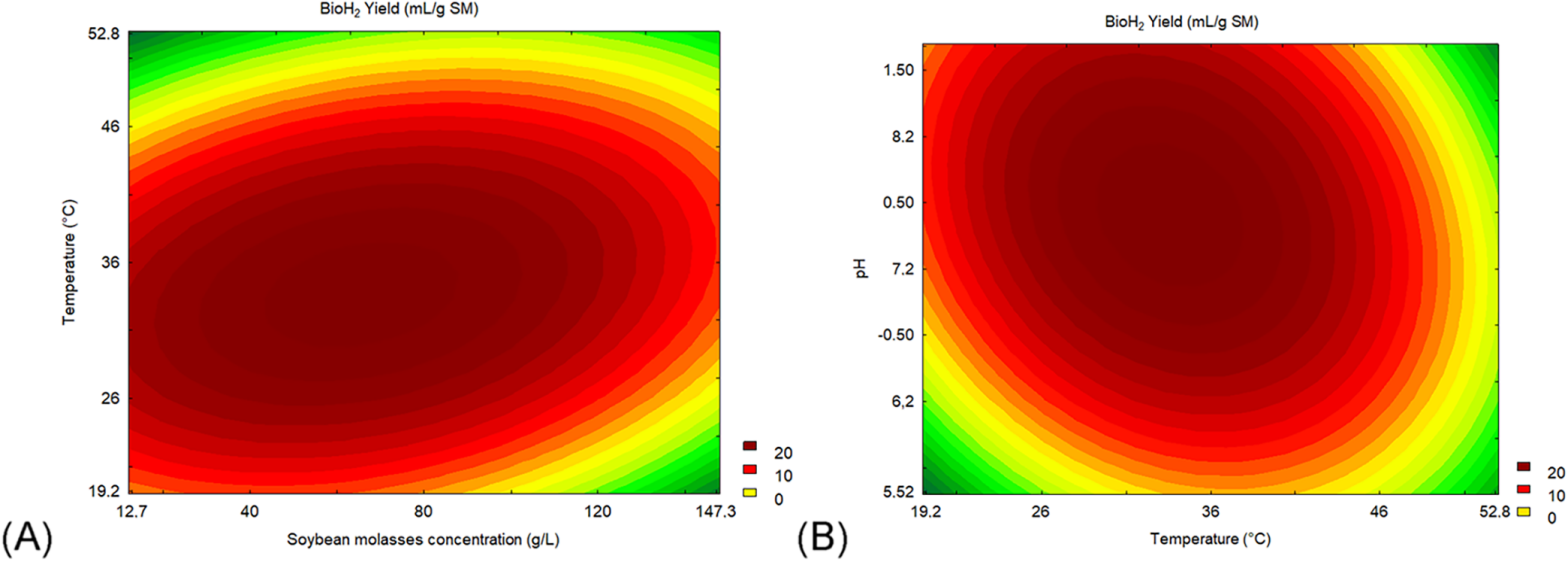
Contour plots of BioH_2_ Yield (mL/g SM); (A) temperature vs soybean molasses concentration (g/L) and (B) pH vs temperature (°C).

The addition of SM enhances bioH_2_ production, with higher yields observed between approximately 28 and 105 g/L. Soybean molasses are rich in simple sugars such as fructose and glucose, as well as high amounts of sucrose.^8^ Carbohydrates are the preferred substrates used by microorganisms in dark fermentation processes, representing the main fraction of biomass that can be converted into bioH_2_. For this reason, many studies have used sugars like glucose, sucrose, and fructose for bioH_2_ production by dark fermentation.^30–33^ Thus, due to the high costs of sugars in their pure form, the use of soybean molasses presents itself as a successful alternative.^34^

The high amounts of protein present in soybean molasses also allow its use as a nitrogen source.^8^ Nitrogen is an essential nutrient for bioH_2_ production, as it is used by bacteria in protein synthesis, nucleic acids, and enzymes, which play an important role in biomass formation and consequently, in bioH_2_ production.^35^

MARTINEZ-BURGOS^36^ reported a significant effect of adding various nitrogen sources (yeast extract, urea, and ammonium sulfate) on bioH_2_ production by a consortium from cassava starch processing wastewater. The highest yields were achieved using yeast extract. However, bioH_2_ production depends on the use of widely available and low-cost substrates, making the supplementation of the medium with high-cost additives economically unfeasible,^37^ as is the case with yeast extract. Thus, supplementation with soybean molasses also presents itself as an alternative to the addition of nitrogen sources.

On the other hand, it was observed that initial concentrations of soybean molasses above 105 g/L lead to a decrease in bioH_2_ production. This decrease may be due to the inhibition of microorganisms caused by the high organic load. Several studies have investigated the ideal concentrations of organic load for bioH_2_ production^38,39^ indicating that this parameter is directly related to bioH_2_ yields.

Temperature is also a significantly important variable for the process as it affects the growth of microorganisms and bioH_2_ production. This parameter affects bacterial growth as it is directly related to cellular reactions and metabolic patterns. The optimal temperature for bioH_2_ production is in the range of ≅27 to 40 °C (Figure 1). The decrease in yields observed at temperatures above the ideal range is explained by the denaturation of microbial enzymes, leading to cell death.^40^ Microorganisms belonging to the Class *Clostridia*, predominant in the consortium used in this work, are mesophilic and have optimal growth conditions at temperatures between 20–40 °C.^41^

Studies using pure strains of *Clostridium* or consortia in which this genus was predominant indicated optimal temperatures around 35 and 40 °C.^42–44^ Furthermore, operating reactors within this temperature range is less costly than in thermophilic and hyperthermophilic ranges.^21^

Regarding the pH of the fermentation medium, it presented itself as a significant variable (*p* < 0.1) and plays a crucial role in the growth and activity of bioH_2_-producing microorganisms. This is because pH affects physiological parameters in cells, such as internal pH, proton motive force, and membrane potential.^45^ Additionally, pH changes can alter the cell membrane charge, which can impact nutrient absorption, thus affecting enzyme activity in metabolic processes. pH can also influence the metabolic pathway used for bioH_2_ production, affecting the formation of byproducts such as organic acids.^46^

In this study, the initial pH of the medium composed of soybean molasses and lysogoma was optimized without the addition of buffers. The optimal initial pH for bioH_2_ production in this study is in the range of ≅6.8 to ≅8.4 (Figure 1).

Studies using pure strains of *Clostridium* or consortia predominantly composed of this genus have reported varying optimal pH values in this range, such as in the work of Luo et al.,^41^ where the optimized initial pH was 7.5. Similarly, JUNGHARE and SUBUDHI^47^ reported an optimal initial pH of 8.0, also using *C. butyricum* as the inoculum. Likewise, Li and Chen^48^ reported an optimal growth range at pH ranging from 7.0 to 7.5 for fermentations using *C. butyricum*.

Overall, studies on bioH_2_ production from various microorganisms including pure strains or consortia indicate a wide range of optimal pH values, from acidic pH (5.5) to basic pH (8.0).^23,47^ Therefore, experimental designs should be specifically tailored to optimize pH for each bioH_2_ production project.

### 3.3 Experimental Validation of the Mathematical Model

The mathematical model constructed was used to maximize bioH_2_ production by determining the optimal points of g/L of soybean molasses, temperature, and pH. The experimental conditions, predicted results, and experimental results can be observed in Table 5. No statistical differences were demonstrated by the Tukey test (*p* < 0.05) between the predicted and experimental results, confirming the robustness of the mathematical model.

**Table 5:** Optimal Conditions to Produce BioH_2_ According to the Model

concentration of soybean molasses (g/L)	temperature (°C)	pH	expected bioH_2_ (L/L medium)	experimental bioH_2_ (L/L medium)
64.4	32.9	7.6	1.55 ± 0.9	1.6 ± 0.21

The production of bioH_2_ resulted in a maximum yield of 1.6 L/L of culture medium after 48 h of fermentation, as predicted by the model. The biogas resulting from dark fermentation was composed of H_2_ (≅57.14%) and CO_2_ (≅42.86%) without traces of CH_4_. Using an isolated strain identified as *C. butyricum*, Wang et al.^49^ obtained a production of 1.8 L of bioH_2_/L of the culture medium, composed of 10 g/L of maltose after 23 h of fermentation^23^ obtained a maximized production of 0.71 L of bioH_2_/L of the culture medium using pretreated rice straw as the substrate through a combination of acid hydrolysis and a microbial consortium as inoculum. Using glycerol and wastewater from cassava processing as substrate and thermally pretreated inoculum from anaerobic reactor sludge, Meier et al.^50^ obtained a production of 0.86 L of bioH_2_/L of the culture medium. Rosa et al.^51^ reported a production of 4.62 L H_2_/L of the culture medium after 8 days of fermentation, using hydrolyzed and supplemented palm oil mill effluent (POME) with 7.5 g/L of sucrose and fermented using the strain *Clostridium beijerinckii*.

### 3.4 Production of bioH_2_ and Organic Acids under Optimal Conditions

An adaptation phase of the inoculum occurred until the 26th hours of kinetics (Figure 2). Subsequently, the exponential phase of bioH_2_ production began (26–36 h), remaining stable after this period.

**Figure 2:**
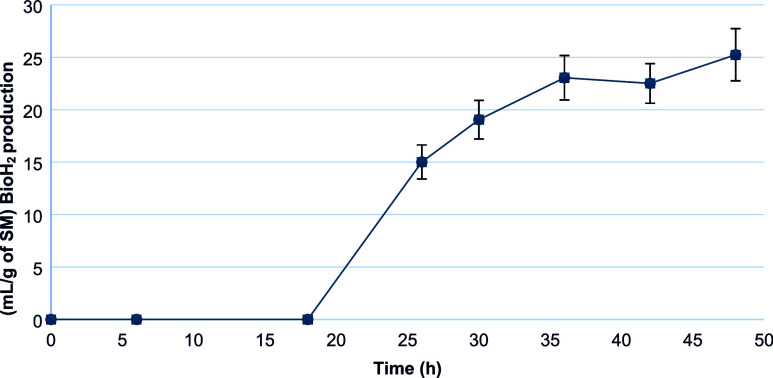
Accumulated production of bioH_2_.

The sugar consumption analyses (Figure 3) predominantly indicate the consumption of sucrose. Song et al.^52^ demonstrated in their study that a newly isolated strain identified as *C. butyricum* could utilize a wide variety of carbohydrates such as sucrose, glucose, and starch as substrates for dark fermentation, with a higher yield using sucrose.

**Figure 3:**
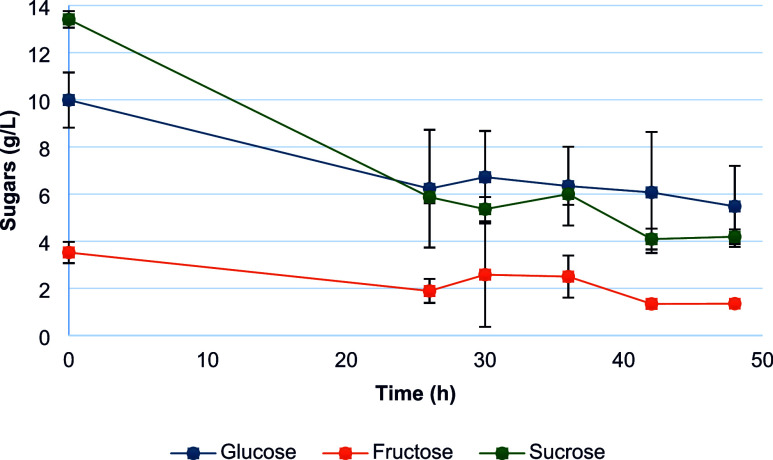
Sugar consumption by the consortium throughout fermentation in a medium composed of lysogoma and soybean molasses.

Litti et al.^53^ showed that the *C. butyricum* strain could utilize a wide variety of sugars including mono-, di-, and trisaccharides, belonging to both hexoses and pentoses: maltose, glucose, mannose, fructose, lactose, galactose, sucrose, xylose, raffinose, cellobiose, and arabinose. Figure 3 further demonstrates that even glucose and fructose were partially consumed in bioH_2_ production in this study.

Acetic and butyric acids were produced, reaching cumulative rates of 3.76 ± 0.46 g/L and 3.28 ± 0.6 g/L, respectively, at the end of the fermentation process (Figure 4). However, it is noted that these compounds were already present in the culture medium before the start of fermentation. Acetic acid had an initial quantity of 2.78 ± 0.08 g/L, and butyric acid had 1.03 ± 0.89 g/L. Thus, butyric acid was the most produced volatile fatty acid during the fermentative process.

**Figure 4:**
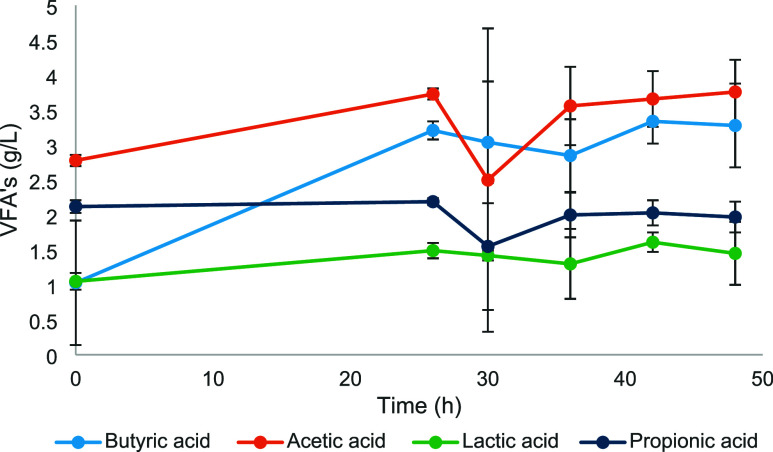
Production of volatile fatty acids (VFAs) throughout fermentation.

Published studies also reported the predominance of acetic and butyric acids in dark fermentation carried out by pure strains of *Clostridium* ^47^ or consortia predominantly composed of the genus in question.^38,40^ Batista et al.,^54^ using the same substrate and inoculum, obtained a production of butyrate (2.5 g/L) and acetate (1.4 g/L) and small amounts of formate (0.4 g/L). Litti et al.^53^ reported the production of acetate (12–34%) and butyrate (27–54%) (47–89% total) through bioH_2_ production from a variety of simple sugars and wastewater from confectionery, wastewater from saccharin beet processing, and cheese whey.

Additionally, if the pH of the culture medium is not controlled, the accumulation of volatile fatty acids leads to a decrease in pH and consequent inhibition of the dark fermentation process.^46^ In this study, pH control was not performed, and a sharp decrease in pH from 7.6 to 5.8 was observed early in the exponential phase (26 h) when bioH_2_ and volatile fatty acids began to be produced, reaching 5.09 at the end of the bioH_2_ production exponential phase (36 h). At this point, bioH_2_ production stabilized, allowing the determination of the end of fermentation, as there was no statistical difference in bioH_2_ production at 36, 42, and 48 h points. The pH drops from 7.0 to 4.5 in the early stages of fermentation due to the production of volatile fatty acids was also observed in the work of Argun et al.^55^ Similarly, Luo et al.^41^ observed a decrease in pH from 7.5 to 4.5 after 18 h of fermentation, using food waste as a substrate and pretreated inoculum from an anaerobic reactor.

Fermentations without pH control significantly affect bioH_2_ production. Therefore, pH control through the addition of buffers such as KH_2_PO_4_ can be performed to maintain the pH of the medium at its optimal condition, resulting in increased bioH_2_ yields.^44,56^

One sustainable solution could be to explore substrates with higher pH or alkalinity to balance the system.^57^ Tenca et al.^58^ investigated the effect of mixing ratios of fruit and vegetable waste with pig manure, aiming to enhance bioH_2_ production while maintaining process stability. Their findings highlighted the importance of the endogenous alkalinity provided by pig manure, which helped buffer the system and mitigate acidification–a common challenge in dark fermentation processes. CHOI; AHN^59^ also suggested the use of substrates with inherently higher pH values to replace the use of buffers.

In this particular study, there was no statistical difference between initial and final COD values (Tukey, *p* < 0.05), which remained constant throughout the fermentation process (≅72.000 mg/L). As mentioned earlier, dark fermentation processes may not result in significant COD removal due to the incomplete oxidation of organic matter, leading to the formation of secondary metabolites trapped in the dark fermentation culture medium.

Elbeshbishy et al.^60^ studied the effect of various pretreatments on food waste to assess their effects on increasing bioH_2_ yields. All experiments indicated low COD removal, ranging from 7% to 14%.

Thus, despite the benefits of dark fermentation, bioH_2_ yields are still limited by the accumulation of volatile fatty acids, requiring treatment of dark fermentation effluent before disposal.^45^ Two-stage fermentation processes have been proposed to utilize the remaining organic matter in dark fermentation effluents, increasing bioH_2_ yields and reducing COD.^12^ This second stage can be performed through photofermentation^61^ or microbial electrolysis cells,^62^ or by using anaerobic digestion processes for methane production.^63^

### 3.5 Molecular Identification of Microbial Consortium

The metagenomic analysis of the consortium (SisGen A9A19CA) composition revealed microorganisms belonging to the *Firmicutes* Phylum, of which about 99.95% are from the *Clostridia* Class (Figure 5). The results obtained demonstrate the effectiveness of the pretreatment method used in the inoculum employed in this study. Furthermore, species-level identification indicated that 99.1% of *Clostridia* Class microorganisms present in the consortium belong to the *C. butyricum* group. Despite the predominance of *Clostridia*, the consortium used in this study is also composed of a wide variety of microbial families in small proportions. The use of consortia in bioH_2_ production is favored over the use of pure strains due to better substrate utilization and robustness to fluctuations in environmental conditions.^45^ The presence of classes of microorganisms comprising not only strict anaerobes like *Clostridia* but also facultative anaerobes like Bacilli, *Alphaproteobacteria*, and *Bacteroidia* can also be observed.

**Figure 5:**
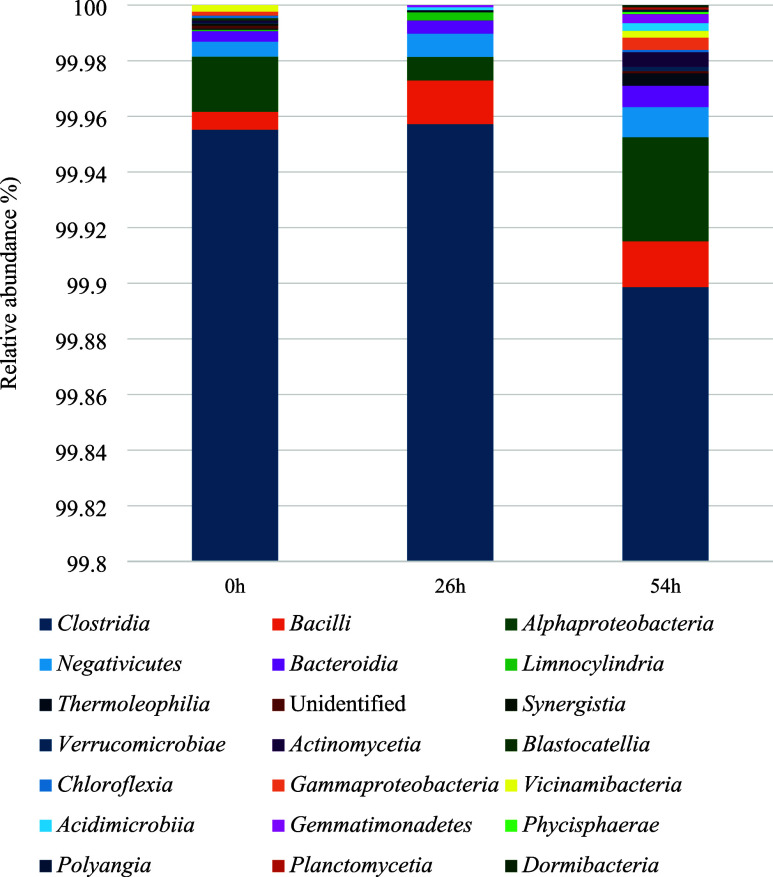
Profile of the relative abundance of families in the consortium throughout the fermentation.

These results are supported by the study of Luo et al.,^41^ where the effects of aeration, acid, base, heat, heat + CO_2_, free nitrous acid, bromoethanesulfonate, and electric shock were tested on the pretreatment of sludge from an anaerobic reactor for subsequent use in bioH_2_ production. All pretreatments reported by the author resulted in an enrichment of 63%–90% of microorganisms belonging to the *Firmicutes* Phylum, compared to untreated raw inoculum (29%), reaching above 90% for heat-treated and heat + CO_2_-treated inoculum.

Although the highest bioH_2_ yields are obtained from strict anaerobic microorganisms, these are very sensitive to low amounts of oxygen (O_2_) and, therefore, their production must be carried out in a completely O_2_-free atmosphere ensured by purging with gases such as nitrogen (N_2_).^64^ Therefore, the use of consortia also composed of facultative anaerobic microorganisms can be a beneficial strategy, as these microorganisms are less sensitive to O_2_ and, in its presence, perform aerobic respiration, rapidly depleting the O_2_ from the fermentative environment, thus favoring higher bioH_2_ yields by strict anaerobes such as *Clostridium*.^46^

The interaction of bacteria that compose a microbial consortium is quite complex, and studies indicate that cell-to-cell communication promotes a phenomenon of cooperation, resulting not only in better substrate utilization but also in greater robustness concerning environmental fluctuations,^46^ causing the prevalence of microorganisms in the consortium to depend directly on the existence of others.^65^

## 4 Conclusions

The culture medium proposed in this study was composed of soybean lysogoma and soy molasses and optimized using a Central Composite Rotatable Design (CCRD), and the optimal cultivation conditions were obtained through the generated mathematical eq (64.4 g/L of soy molasses, pH 7.6, and 32.9 °C).The optimization resulted in the production of biogas composed of bioH_2_ (≅57.14%) and CO_2_ (≅42.86%), achieving a yield of about 1.6 L of bioH_2_/L of culture medium after 48 h of fermentation, demonstrating the potential of the explored culture medium. The consortium obtained was predominantly composed of microorganisms from the *Clostridia* class, reflecting the success of employing thermal pretreatment of sludge from an anaerobic reactor, resulting in the inhibition of methanogenic microorganisms and enrichment of the consortium with spores of microorganisms belonging to the *Clostridia* class, which presented a relative abundance of 99.8% in the initial consortium. BioH_2_ was produced exponentially during 26–36 h of fermentation, preferentially consuming sucrose and resulting in the production of acetic and butyric acids as process byproducts. The incomplete utilization of sugars in the culture medium and the production of VFAs as fermentation byproducts resulted in the maintenance of the COD of the culture medium, which remained constant from the beginning to the end of fermentation. Sustainability in the production of biohydrogen from waste materials is a promising approach that addresses energy needs and waste management challenges. Future studies should evaluate the scalability of the process, including performance analyses in larger-scale reactors, integration of molasses and lysogoma into continuous industrial routes, and the application of life-cycle assessment (LCA) approaches to quantify the environmental benefits and the real potential for incorporating this technology into biorefineries.
